# Supplementary material for: Evidence supported by Mendelian randomization: impact on inflammatory factors in knee osteoarthritis
Source: Front Med (Lausanne). 2024 May 28;11:1382836. doi: 10.3389/fmed.2024.1382836 (PMC11165061; doi:10.3389/fmed.2024.1382836)
Supplement: Supplementary file 1 [file Data_Sheet_1.ZIP › data/GCST90274824_5e6/824.leaveoneout.pdf]

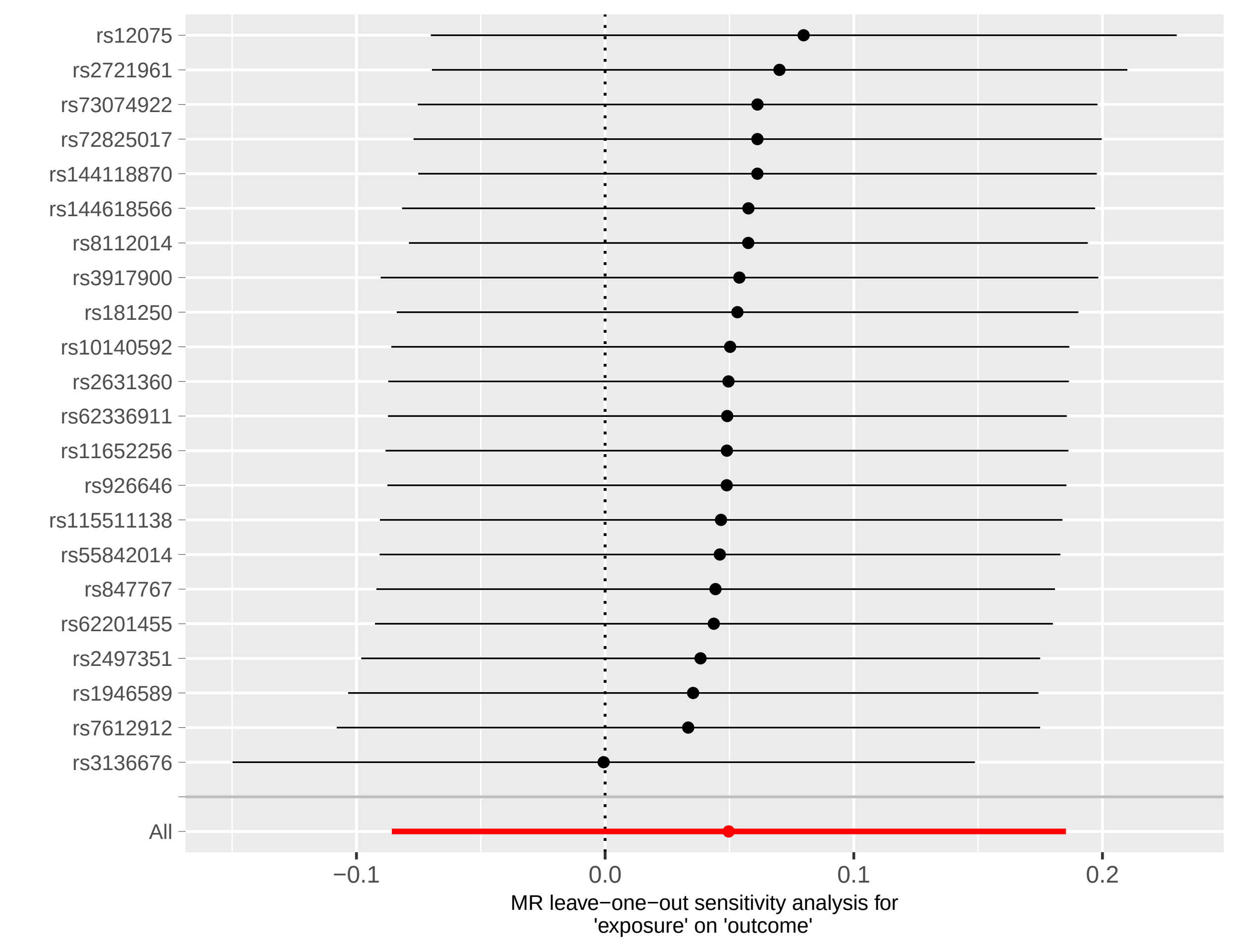

rs12075
rs2721961
rs73074922
rs72825017
rs144118870
rs144618566
rs8112014
rs3917900
rs181250
rs10140592
rs2631360
rs62336911
rs11652256
rs926646
rs115511138
rs55842014
rs847767
rs62201455
rs2497351
rs1946589
rs7612912
rs3136676
All
−0.1
0.0
0.1
0.2
MR leave−one−out sensitivity analysis for
'exposure' on 'outcome'
